# The Ascidian-Derived Metabolites with Antimicrobial Properties

**DOI:** 10.3390/antibiotics9080510

**Published:** 2020-08-13

**Authors:** Marcello Casertano, Marialuisa Menna, Concetta Imperatore

**Affiliations:** Department of Pharmacy, University of Naples “Federico II”, Via D. Montesano 49, 80131 Napoli, Italy; marcello.casertano@unina.it (M.C.); cimperat@unina.it (C.I.)

**Keywords:** ascidian, antibacterial, antimicrobial, antiviral, marine natural products, ascidian-associated microorganisms

## Abstract

Among the sub-phylum of Tunicate, ascidians represent the most abundant class of marine invertebrates, with 3000 species by heterogeneous habitat, that is, from shallow water to deep sea, already reported. The chemistry of these sessile filter-feeding organisms is an attractive reservoir of varied and peculiar bioactive compounds. Most secondary metabolites isolated from ascidians stand out for their potential as putative therapeutic agents in the treatment of several illnesses like microbial infections. In this review, we present and discuss the antibacterial activity shown by the main groups of ascidian-derived products, such as sulfur-containing compounds, meroterpenes, alkaloids, peptides, furanones, and their derivatives. Moreover, the direct evidence of a symbiotic association between marine ascidians and microorganisms shed light on the real producers of many extremely potent marine natural compounds. Hence, we also report the antibacterial potential, joined to antifungal and antiviral activity, of metabolites isolated from ascidian-associate microorganisms by culture-dependent methods.

## 1. Introduction

Antimicrobial agents (including antibiotic, antiviral, antifungal, and antiprotozoal drugs) certainly are critical tools for the treatment of infectious diseases, and many feats of modern medicine and surgery also are determined by the availability of effective antibiotics. Unfortunately, many microorganisms have developed several and different mechanisms of drug-resistance, mainly owing to the misuse and overdose of antimicrobial drugs. Indeed, many marketed anti-infective drugs are losing their efficacy at a rapid pace, and antimicrobial resistance has emerged as one of the major problems that the public health has had to face in the 21st century [1,2]. It has been estimated that, by the year 2050, ten million people will die per year worldwide as a direct consequence of antimicrobial resistance [3]. Resistant infections are on the rise globally, and all countries are affected. In the United States, about 3 million people get an antibiotic-resistant infection each year, with more than 35,000 deaths. On the other hand, in Europe antibiotic resistant causes ~33,000 deaths annually. As for children and neonates, they are disproportionately affected by antibiotic resistant infections, particularly in the poorest regions worldwide. For example, approximatively 30% of neonates affected by sepsis die as a result of the ineffectiveness of antibiotic treatment [2]. Thus, the need for novel antimicrobial drugs is truly great and, besides the search for vaccines as well as for novel diagnostic tools to understand the resistance mechanisms, considerable importance is given to find new chemical scaffolds for antibiotic development. However, where we can find new lead compounds with unprecedented activities?

Natural products, which are the result of the countless possible interactions among the millions of diverse terrestrial and marine species existing worldwide, are evolutionarily optimized as intrinsic drug-like molecules and play an increasingly crucial role in drug discovery owing to their great chemical diversity. The wide chemical space occupied by the secondary metabolites of living organisms and microorganisms is an authentic example of how combinatorial chemistry has been performed by nature for thousands of years, giving a real arsenal of drug lead candidates [4]. The marine environment covers 71% of the Earth’s surface and comprises 50–80% of the total global biodiversity, which translates into an enormous chemical diversity [5]; every year, hundreds of novel compounds are isolated and identified, especially from marine invertebrates [6,7,8,9,10,11,12,13,14,15,16,17]. In fact, these species are sessile or slow moving and soft bodied; although they commonly host microorganisms, they lack the sophisticated adaptable immune systems seen in vertebrates. Nevertheless, they thrive and keep themselves free of infections, which means that they rely entirely on their innate immune system. It has been widely proven that, as a part of their effort for survival, marine invertebrates have developed biosynthetic pathways for production of a wealth of secondary metabolites to be used as chemical defense and communication. In this evolutionary process, their symbiotic association with microorganisms plays a key role [18].

**Figure 1:**
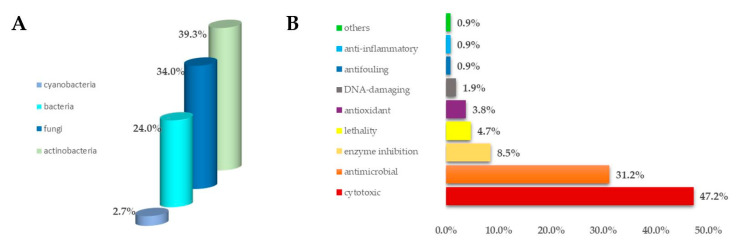
(**A**) Percentage distribution of microorganisms paired to ascidians from which marine metabolites derive; (**B**) bioactivities of secondary metabolites isolated from ascidian-associated microorganisms described as percentage distribution.

Among the marine invertebrates, ascidians are the most closely related to humans; they constitute the biggest and most varied class of the sub-phylum Tunicata, comprising about 3000 described species [19]. Ascidians have been shown to be an exceptionally important source of natural products with biomedical and pharmaceutical applications, especially, but not exclusively in cancer [10,11,20,21,22]. Furthermore, ascidians harbour a great microbial community (including bacteria, actinobacteria, cyanobacteria, and fungi; Figure 1A), which represents an additional source of natural products, many of which are extremely potent and mainly cytotoxic and antimicrobial, but also antioxidant, anti-inflammatory, and many others (Figure 1B) [23]. Indeed, it has been proven that about 8% of the bioactive molecules isolated from ascidians derive from symbiotic microorganisms; recently, the development of culture-independent methods allowed to isolate and study the ascidian-associated microorganisms and their involvement in the synthesis of bioactive metabolites isolated from the invertebrates [24].

The aim of the present review is to focus the chemical diversity of the secondary metabolites with antibacterial activity (often joint to antiviral, antifungal, or antiprotozoal effects) isolated from marine ascidians through January 2020. This collection shows that, so far, more than 160 substances have been identified from marine ascidians with this activity, with different chemical structures and, therefore, most likely acting through different mechanisms. Their chemical and pharmacological properties are described herein; the molecules have been classified and grouped on the basis of some their common chemical features, in order to give a rational view of the high potential of ascidians as a source of new chemical scaffolds to be used as antimicrobial leads. Finally, a conspicuous section of the review has been devoted to the recent progress in the study of some ascidians-associated microorganisms and their role in the production of the bioactive metabolites.

## 2. Results and Discussion

### 2.1. Sulfur-Containing Metabolites: Polysulfides and Alkyl Sulfates

**Figure 2:**
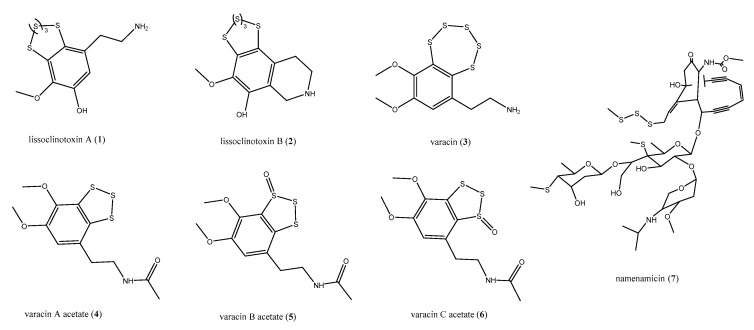
Structures of the antimicrobial polysulfides lissoclinotoxins (**1**–**2**), varacins (**3**–**6**), and namenamicin (**7**).

Generally, natural products contain one or two heteroatoms like nitrogen and/or oxygen, sometimes more than two, whereas a sulfur atom is rarely found in metabolites of marine origin [25,26]. The ascidians belonging to the genus *Lissoclinum* (Didemnidae) are an exceptional source of unique chemical scaffolds, some of them endowed with antiviral activity as well as against mammalian cells [27,28]. Among these metabolites, several structurally intriguing antimicrobial polysulfides have been identified from *Lissoclinum* and *Polycitor* species, active against an array of pathogenic bacteria, fungi, and other infective agents [29,30,31,32]. The bioassay-guided chemical investigation of *L. perforatum* led to the isolation of lissoclinotoxins A (**1**) and B (**2**), (Figure 2) [29,30]. An extensive pharmacological characterization of these polysulfides [30] evidenced that compound **1** was active against *Staphylococcus aureus,* showing very low minimum inhibitory concentration (MIC) values (0.08–0.15 μg/mL), in the same range of those of cefatoxim. On the other hand, when compounds **1** and **2** were tested against *Aeromonas salmonicida* and *Vibrio anguillarium* by the inhibition zones assay, lissoclinotoxin B (**2**) was more potent that **1** against both bacteria, mainly against *A. salmonicida* with an 8 mm inhibition zone when tested at 5 μg with respect to **1**, which showed only 40 mm/30 μg [28]. Compound **1** exhibited further antimicrobial properties; it showed moderate toxic effects against *Trichosporon mentagrophytes* and *Candida albicans* fungal strains, with MIC of 20 and 40 μg/mL, respectively [30], and was also active against a resistant strain of *Plasmodium* isolated from infected erythrocytes. In this latter case, it exhibited a lower IC_50_ value (296 nM) with respect to the commercially available antimalarials chloroquine and quinine (IC_50_ values of 580 and 350, respectively).

In a first study, the antifungal properties of the benzopentathiepin varacin (**3**, Figure 2), isolated from *L. vareau*, were demonstrated against *C. albicans*, with a 14 mm zone of inhibition when tested at 2 μg per disk [31]. However, it was strongly cytotoxic, being 100 times more potent than 5-fluorouracil with an IC_90_ of 0.05 μg/mL against human colon cancer HCT 116; its role in the damage of DNA was proven too [31]. The role played by ascidians-associated microorganisms in the synthesis of their secondary metabolites is sometimes suggested by the discovery of analogous and/or the same compounds in ascidians belonging to different genus and families as well as collected in completely different areas. This is the case of varacin (**3**), which was also recovered from the colonial ascidian *Polycitor* sp. [32] along with three further polysulfides, isolated as acetates (varacin A–C acetates, **4**–**6**, Figure 2). Compounds **3**–**6** were subjected to antibacterial and antifungal assays; as varacin (**3**), the acetates **4**–**6** exhibited strong activity in vitro against *C. albicans.* In addition*,* both varacin (**3**) and the derivatives **4**–**6** were effective against the Gram-positive *Bacillus subtilis,* with **3** being 10-fold more active than compounds **4**–**6.**

*Polysyncraton lithostrotum*, an ascidian of Didemnidae family, was the source of namenamicin (**7**, Figure 2), a compound with an unusual “enediyne warhead” and an *S*-methyl group appended to a sugar moiety [33]. Compound **7** exhibited interesting antibiotic properties, being more potent than penicillin G against *Enterococcus faecium, Klebsiella pneumoniae, C. albicans, Ustilago maydis, Saccharomyces cerevisiae,* and *Neurospora crassa*, with MIC values of 0.03, 0.06, 0.25, 0.004, 0.06, and 0.25 μg/mL, respectively. However, as varacin, namenamicin exerted a strong cytotoxic effect in vitro [33].

**Figure 3:**
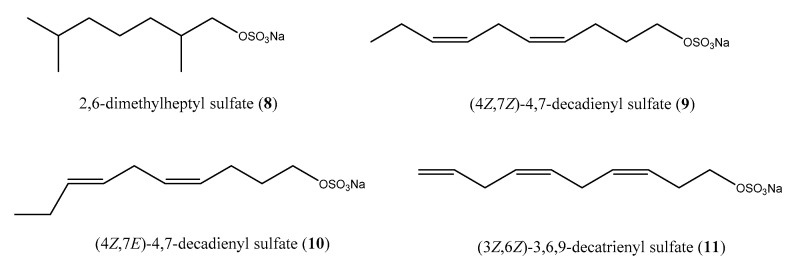
Structures of alkyl and alkenyl sulfates **8**–**11**.

Metabolites endowed with sulfated groups, although rather unusual, occur in marine invertebrates; nevertheless, in the last years, sulfated alkanes and alkenes are increasingly turning out to be common natural products of marine ascidians [34,35]. The chemical analysis of the ascidian *Halocynthia roretzi* allowed the identification of the antimicrobial alkyl sulfates 2,6-dimethylheptyl sulfate (**8**) such as racemate, (4*Z*, 7*Z*)-4,7-decadienyl sulfate (**9**), (4*Z*, 7*E*)-4,7-decadienyl sulfate (**10**), and (3*Z*, 6*Z*)-3,6,9-decatrienyl sulfate (**11**) (Figure 3) active against the Gram-negative marine bacterium *V. alginolyticus* and the fungus *Mortierella ramaniana* [36]. The activity was evaluated by paper disk method by administration of 0.2 mg per disk of each compounds. Moreover, Fusetani and co-workers investigated the occurrence of these compounds in the ascidian tissues. Dissection of *H. roretzi* provided several ascidian tissues (i.e., hepatopancreas, gonad, tunic, body muscle, and hemolymph), which were separately analyzed. Alkyl sulfates **8**–**11** were found only in the hepatopancreas of the organism, underlining that these sulfates may be involved in some physiological processes of ascidian digestive system [36].

### 2.2. Meroterpenes

**Figure 4:**
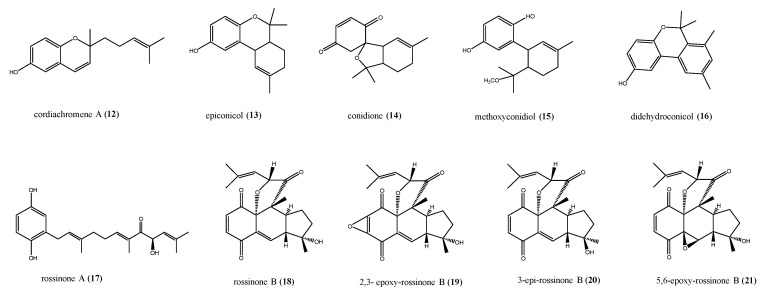
Structures of the geranylhydroquinone derivatives **12**–**16** and of rossinones (**17**–**21**).

Molecules of mixed biosynthesis consisting of quinone/hydroquinone rings bonded to a prenyl portion are commonly called meroterpenes; they have a wide occurrence in nature and are considered “privileged scaffolds” in medicinal chemistry owing to their wide range of bioactivities, often owing to the quinone/hydroquinone moieties undergoing redox cycling [37,38,39,40]. This latter generates an oxidative burst accompanied by a huge and transient amount of reactive oxygen species (ROS), responsible for cellular damage. For this reason, both natural and synthetic prenylated quinones and hydroquinones found extensive application in the field of chemotherapy and cancer-preventive treatment [41,42,43]. A number of antimicrobial prenylated quinones from ascidians have been discovered too. Cordiachromene A (**12**) and epiconicol (**13**) are two geranylhydroquinone derivatives isolated from *Synoicum castellatum*, a species closely allied to the ascidians of genus *Aplidium* (Figure 4) [44]. In fact, naloogues quinoid compounds with a mixed shikimate-mevalonate biogenesis have been found in several *Aplidium* species. Conidione (**14**, Figure 4), with cytotoxic, anti-inflammatory, and even anti-HIV activity, has been isolated from samples of *A. conicum* [45], and all three compounds **12**–**14** have been recovered from *A. densum*, along with methoxyconidiol (**15**) and didehydroconicol (**16**, Figure 4) [38,46]. The antibacterial activity of compounds **12**–**16** was quantitatively determined against the Gram-negative bacterium *E. coli* and the Gram-positive bacterium *Micrococcus luteus.* The tested compounds **12**–**16** did not show any activity against *E. coli*. On the other hand, epiconicol (**13**, MIC = 0.13 mmol), didehydroconicol (**16**, MIC = 0.52 mmol), and cordiachromene A (**12**, MIC = 0.51 mmol) showed weak activity against *M. luteus*. Studies were conducted on the active meroterpenes (**12**, **13**, and **16**) in order to distinguish for bacteriostatic or bactericidal effect. In the case of **12**, as bacterial growth did not restart, an aliquot of the bacteria suspension treated with this compound was placed on a solid nutritive medium; compound **12** caused bacterial killing and could be considered as bactericidal [46,47]. Instead, **13** and **16** only inhibited the bacterial growth in the solid medium, thereby showing a bacteriostatic effect [46].

The triprenylated hydroquinones, rossinones A and B (**17** and **18**, Figure 4), possessing a rare linear fused 6,6,5-ring core, were isolated during a campaign to collect colonial ascidians of genus Aplidium in Ross Sea [48]. Both compounds **17** and **18** exhibited antimicrobial activity against the bacterium *B. subtilis* and the fungi *Trichophyton mentagrophytes* (3–6 mm excess radius at 60 μg/disk) and an antiviral effect (2 μg/disk) toward DNA Herpes simplex virus 1 (HSV-1) and RNA Poliovirus-1 (PV-1). Moreover, only rossinone B (**18**) had antiproliferative activity inhibiting SH-SY5Y neuroblastoma (IC_50_ = 1.6 μM) and P388 murine leukaemia (IC_50_ = 0.084 μM) cell lines [48]. Rossinone B (**18**) was later re-isolated from Antarctic samples of *A. fuegiense,* together with several minor analogues, 2,3-epoxy-rossinone B (**19**), 3-epi-rossinone B (**20**), and 5,6-epoxy-rossinone B (**21**, Figure 4) [49,50]. This study revealed, through chemical analysis of the dissected parts of the organism separately, that these metabolites were concentrated in the colony inner part (viscera) and only a small amount of compound **18** was recovered in the external part (tunic) [49]. Generally, in the colonial ascidians, the defensive chemicals are concentrated in special cells in the outer tunic, while other intermediate products remain in the inner producing tissues [50]. This could explain the distribution observed for the rossinone compounds in *A. fuegiense,* where rossinone B could be the major and most active defensive metabolite, and thus present mainly in the inner tissues, but also in the tunic in small amounts. On the contrary, the minor compounds **19**–**21**, only present in the internal part of the colony, are presumably precursors or, alternatively, could derive from symbiotic microbes [50]. No data are reported about the antimicrobial properties of compounds **19**–**21**. Rossinone B (**18**) was also tested on cultures of an unidentified sympatric Antarctic marine bacterium. Interestingly, although it was active towards cosmopolitan bacterial strains [48], it revealed no activity in this assay [50].

### 2.3. Saturated and Unsaturated Amino Alcohols

**Figure 5:**
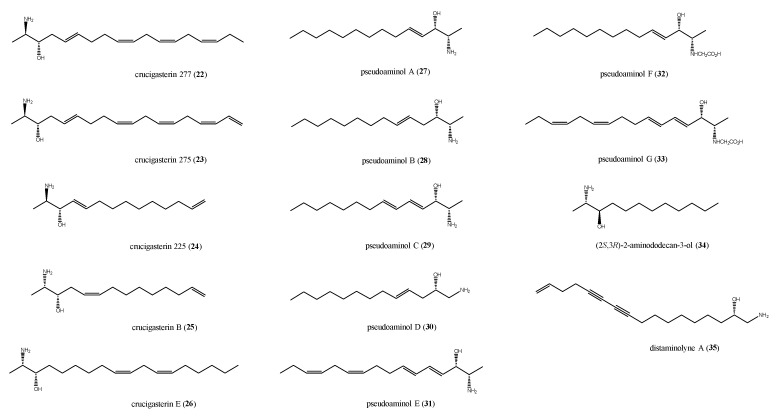
Structures of the amino alcohols crucigasterins (**22**–**26**), pseudoaminols (**27**–**33**), (2*S*, 3*R*)-2-aminododecan-3-ol (**34**), and distaminolyne A (**35**).

The significant antimicrobial and cytotoxic potential of marine derived saturated and unsaturated amino alcohols stimulated a great interest toward these metabolites. The first examples of amino alcohols with antimicrobial activity from ascidians are the crucigasterins, firstly isolated from the Mediterranean ascidian *Pseudodistoma crucigaster* [51,52]. The selected crucigasterins described in this review (compounds **22**–**26**, Figure 5) all feature a 2-amino-3-alcohol terminal group of an unsaturated alkyl chain endowed with mixed *E*/*Z* geometries of the double bonds. They differ in the carbon chain length and the oxidation degree; compounds **25** and **26** possess the 1,2-*syn* relative configuration at the amino alcohol moiety whereas for compounds **22**–**24**, the 1,2-*anti* configuration is reported [51,52].

Biosynthetic studies strongly suggested that these derivatives are produced starting from both fatty acids and either L- or d-alanine. The 2*R* absolute configuration in compounds 22–24 implied that they should derive from d-alanine, whereas crucigasterins B and E are L-alanine derivatives [53,54]. Crucigasterins 22–24 showed similar effects against *B. subtilis,* with inhibition zones of 10, 9, and 9 mm, respectively, at 10 μg/disk each. Slight differences were indeed observed for the antifungal activity when compounds 22–24 were tested against the yeast *S. cerevisiae* at 5 μg/disk; compound 22 was the most active in the series, with a 12 mm inhibition zone, whereas crucigasterins 23 and 24 induced 15 mm and 17 mm inhibition, respectively [52]. Crucigasterin B (26) was selectively active against the Gram-negative bacterium *E. coli* at 100 μg/mL and did not exhibit any effect against the yeast *C. albicans*. Crucigasterin B (25), instead showing both antibacterial and antifungal properties at 50 μg/mL.

Pseudoaminols A–G (**27**–**33**, Figure 5), obtained from a *Pseudistoma* sp. collected along the Korean coasts [55], possess structures closely related to those of crucigasterins. Pseudoaminols F (**32**) and G (**33**) are the first report of glycine-derived amino alcohols, as attested by the presence, in their structures, of a *N*-carboxymethyl group [55]. All compounds **27**–**33** were screened for their antibacterial activity against strains of Gram-positive and Gram-negative bacteria. Pseudoaminols A and B (**27** and **28**) displayed significant activity against all the tested bacterial strains except for *E. coli* (Table 1); the effects were similar for the two compounds except for *Salmonella typhimurium*, where **27** was more potent than **28** (Table 1). Both pseudoaminols A and B were slightly cytotoxic against pulmonary adenocarcinoma A549 and chronic myeloid leukaemia K562 cell lines, too [55].

The reduction of polarity and/or basicity of the amino and hydroxyl groups, for example, with the introduction of a *N*-carboxymethyl substituent, as in the inactive compounds 32 and 33, emphasizes the crucial role played by these functions in the bioactivity either for electronic effects or steric hindrance [55]. Moreover, the above data suggest that the number of double bonds in the amino alcohols alkyl chain strongly affects the antimicrobial activity, too; if within the group of pseudoaminols, it seems that, as the number of double bonds increases, the activity decreases [55], and the saturated amino alcohol (2*S*, 3*R*)-2-aminododecan-3-ol (34, Figure 5), isolated from a sample of *Clavelina oblonga* when tested on both oxacillin-resistant and sensitive strain of *S. aureus*, was inactive. It showed indeed a strong antifungal activity against *C. albicans* (MIC = 0.7 ± 0.05 μg/mL) and a moderate effect against *C. glabrata* (MIC = 30.0 μg/mL) [56].

The one example of antimicrobial acetylenic amino alcohol is distaminolyne A (35) from *P. opacum* (Figure 5) [57]. Compound 35 showed MIC values of 32 μg/mL (85 μM) against *S. aureus* and *Mycobacterium tuberculosis*, and of 64 μg/mL (170 μM) against *E. coli*. Using microbroth dilution assay, a bactericidal effect was demonstrated for the amino alcohol 35, with a minimum bactericidal concentration (MBC) of 32 μg/mL against *S. aureus*, of 64 μg/mL against *M. tuberculosis*, and MBC >128 μg/mL against *E. coli*.

### 2.4. Spiroketals

**Figure 6:**
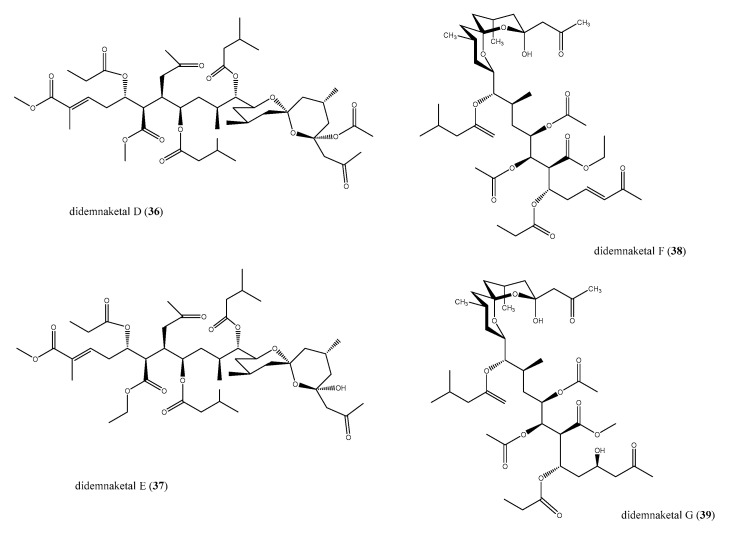
Structures of didemnaketals D–G (**36**–**39**).

The spiroketal moiety widely occurs in bioactive natural products [58,59,60,61]. Within the marine environment, *Didemnum* ascidian species has been the source of unique didemnaketals [62,63,64,65], never previously isolated by other marine species. Didemnaketals D and E (**36** and **37**) featuring a spiroketal/ketal (**36**) or a spiroketal/hemiketal moiety (**37**), as well as didemnaketals F (**38**) and G (**39**) in which a methyl ketone moiety replaced the terminal ester present in **36** and **37**, were tested for their antimicrobic activity (Figure 6).

Didemnaketals D (**36**) and E (**37**) were not effective as antifungal and moderately active against *S. aureus* and *B. subtilis*, with an 11 mm of inhibition zone each [64]. Didemnaketals F (**38**) and G (**39**) were active against both *E. coli* and *C. albicans*; **38** displayed a stronger antimicrobial activity, showing 20 and 24 mm inhibition zones, respectively, at 100 μg/disc (**39** was moderately active against these pathogens with 7 and 17 mm of inhibition zones, respectively, at the same concentration) [65]. Didemnaketals displayed further bioactivities, such as kinases inhibition [64] and cytotoxic properties [65].

### 2.5. Peptides and Peptide-Like Structures

Antimicrobial peptides (AMPs) of marine origin have recently attracted great attention of the scientists for their numerous bioactivities and applications [66,67,68]. Generally, they are rather small peptides (<60 amino acids) with various anti-infective activities against different microorganisms, including Gram-positive and Gram-negative bacteria, fungi, viruses, and parasites [69]; some of them possess anticancer and immunomodulating activities [68]. Marine AMPs represent for the host organisms the first line of defense against invading microbes, and it has been hypothesized that AMPs’ antimicrobial activity is based on their initial electrostatic interaction with the negatively-charged surface of the bacteria [66,70]. Despite the chemical diversity of marine AMPs, a broad classification has been proposed, based on some key structural features and biochemical characteristics shared by most AMPs. They have thus been divided into four general categories, namely, (a) linear α-helical peptides; (b) linear or helical peptides with abundance of one amino acid (proline, tryptophan, histidine, or glycine rich peptides); (c) peptides forming hairpin-like β-sheet or α-helical/β-sheet mixed structures stabilized by intramolecular disulfide bonding; and (d) cyclic peptides [68]. Several AMPs have been isolated from ascidians, all belonging to the linear α-helical peptides category [68]. The solitary ascidian *Styela clava* has been the source of two distinct families of α-helical AMPs, the styelins, and the clavanins, small peptides (2–4 kDa) that were identified from the blood cells (hemocytes) of the ascidian [71,72,73]. The styelins are phenylalanine-rich peptides with 32 amino acid residues, whereas the clavanins are histidine-rich polypeptides with cationic properties with 23 amino acid residues and C-terminal amidation [71,72,73,74]. Styelins killed the marine bacteria *Psychrobacter immobilis* and *Planococcus citreus* in media containing NaCl (0.4 mM); styelins A and B showed also significant activity (MIC ≤ 0.5 μM) against both Gram-negative (*E. coli*, *S. typhimurium*, *P. aeruginosa*) and Gram-positive (*Listeria monocytogenes*, *E. faecium*, *S. aureus)* bacteria [71,72]. Styelin D also was active against Gram-negative and Gram-positive bacteria and was hemolytic and quite cytotoxic to eukaryotic cells [73].

In clavanins A–E, 18 of the 23 amino acid residues are identical, and 4 or 5 are phenylalanine residues. Clavanins were active against infections by fungi and Gram-positive (including multi-resistant *S. aureus* (MRSA)) and Gram-negative bacteria. It has been reported that, when clavelin A is used in a nanoparticle formulation, it shows an increase in its antimicrobial activity and has the potential to be used to treat polymicrobial sepsis infections caused by *S. aureu*s, *E. coli*, *P. aeruginosa*, and/or *K. pneumoniae* [75]. In vivo sepsis bioassays, performed using C57BL6 mice strain inoculated with a polymicrobial suspension, led to a 100% survival rate under sub-lethal sepsis assays and 40% under lethal sepsis assays in the presence of nanoformulated clavanin A [75]. The clavanins showed optimal antibacterial efficacy at acidic pH (pH = 5.5), unlike that of styelins, which demonstrated a broad pH optimum in killing bacteria [73].

Halocyntin and papillosin, two cationic AMPs isolated from the hemocytes of a Mediterranean Sea collection of *H. papillosa* showed strong activity against a panel of both Gram-positive and Gram-negative bacteria [76]. Papillosin (range of activity: 0.05 μM < MBC < 6.25 μM) was eight times more active than halocyntin (range of activity: 0.39 μM < MBC < 50 μM); for both compounds, a low protein binding potential was evidenced, indicating their ability to interact selectively with the lipids of the microbial membrane and not with proteins [76].

Two cysteine-containing α-helical AMPs have been isolated from *H. aurantium,* the homodimer dicynthaurin [77], composed of two 30-residue monomers without any sequence homology to other marine ascidian peptides, and the heterodimer halocidin [78], composed of two subunits containing 18 and 15 amino acid residues that are linked by a single disulfide bond. Dicynthaurin was active against Gram-positive (*M. luteus*, *L. monocytogenes*, *S. aureus*) and Gram-negative bacteria (*E. coli, P. aeruginosa*) at 140 μg/mL (MIC). Furthermore, it possesses antibiotic activity optimal at NaCl concentrations below 100 mM, suggesting that its activities may take place intracellularly rather than extracellularly. Halocidin was shown to be effective against two antibiotic-resistant bacteria, *S. aureus* and *P. aeruginosa* [78], and demonstrated potent antifungal activity, too (MIC = 1–4 mg/mL) [79]. It is to be noted that the 18-monomer subunit is more active than the heterodimer or the 15-monomer against MRSA and multi-drug resistant *P. aeruginosa* [80]. Very recently, novel AMPs, turgencins A and B, along with some their oxidized derivatives have been identified from the Arctic ascidian *Synoicum turgens*, and they represent the first cysteine-rich AMPs isolated from ascidians [81]. All turgecins showed antibacterial activity against both Gram-negative and Gram-positive bacterial strains, but to a different extent. Turgencin AMox1 is the most potent compound in the series, with MICs values of 0.4 μM against both *Corynebacterium glutamicum* and *B. subtilis*, and of 0.8 μM against *E. coli*. Turgencin AMox1 was also cytotoxic, but the MIC against the most sensitive bacteria is still 12 times lower than the IC_50_ against the non-malignant cell line [81].

**Figure 7:**
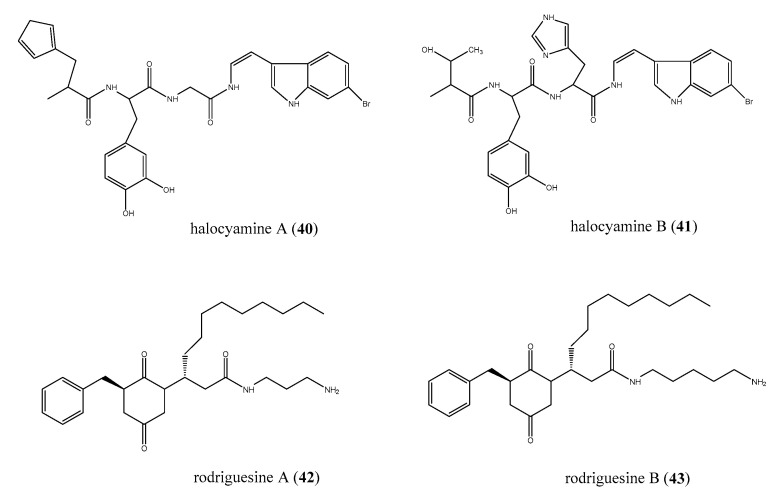
Structures of the peptide-like compounds halocyamines (**40** and **41**), and of rodriguesines A and B (**42** and **43**).

Peptide-like compounds have also been recovered from marine ascidians, some of them belonging to the tunichrome family, a class of reducing 3,4-dihydroxyphenylalanine (DOPA)-containing peptides present in the blood cells of ascidians [82]. Unusual members of this group are halocyamines A (40) and B (41), two tetrapeptide-like compounds containing L- DOPA and a 6-bromoindole DOPA (Figure 7), isolated from the ascidian *H. roretzi* collected in Mutsu Bay, Japan [83,84]. Biological evaluation of halocyamines A and B showed a wide range of activities, including growth inhibition of Gram-positive bacteria, Gram-negative marine bacteria, fungi, and fish RNA viruses [85]. However, when halocyamine A (40) was evaluated against a panel of Gram-positive (*S. aureus, S. intermedius*), Gram-negative (*P. aeruginosa E. coli* ATCC*, E. faecalis*), and marine Gram-negative bacteria *(V. harveyi, V. alginolyticus and Listonella anguillarum*), it showed a modest antibacterial activity and only against *P. aeruginosa* and *E. faecalis* (for both MIC = 100 μM) and *V. harveyi* (IC_50_ = 129 μM).

From a *Didemnum* sp., two modified diketopiperazines, rodriguesines A and B (42 and 43, Figure 7), were isolates as a mixture of inseparable homologues [86]. Both 42 and 43 displayed moderate antibiotic activity against most of the tested pathogen except against *P. aeruginosa* P1 (MIC = 4.3 μg/mL). Interestingly, the mixture of diketopiperazines was proven to be more active against antibiotic-resistant strains than against standard American Type Culture Collection (ATCC) or National Collection of Type Culture (NCTC) strains and also inhibited both oral streptococci and pathogenic bacteria. It has also been reported that rodriguesines were able to inhibit *S. mutans* biofilm formation, downregulating the expression of the bacterium virulence genes gtfB, gtfC, and gbpB without affecting the expression of the house-keeping genes groEL and 16S [87].

### 2.6. Alkaloid Structures

**Figure 8:**
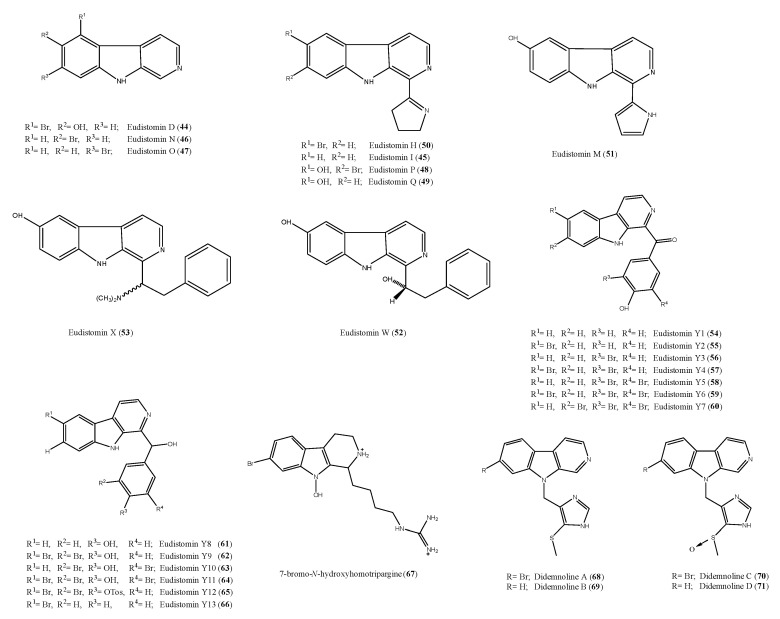
Structures of the β-carbolines alkaloids eudistomins (**44**–**66**), 7-Bromo-*N*-hydroxyhomotrypargine (**67**), and didemnolines A–D (**68**–**71**).

Ascidians are known to be a rich source of bioactive alkaloids, many of them with antimicrobial properties, suggesting their potential value as lead structures for the development of new antibiotics [88,89,90]. Among these, β-carbolines form a large group of tryptophan-derived metabolites frequently isolated from ascidians belonging to several taxonomical groups, mainly from *Eudistoma* species [57,91,92,93]. Eudistomins and didemnolines are members of this family of metabolites and some of them exhibited interesting antimicrobial activity. Several eudistomins were obtained from the extract of the colonial Caribbean ascidian *E. olivaceum.* Eudistomins D, I, N, O, P, and Q (**44**–**49**, Figure 8) exhibited modest activity against *B. subtilis* [94,95]. However, for eudistomins I, N, O (**45**–**47**), H (**50**), and M (**51**) (Figure 8), previously reported as “moderately active” against viruses and bacteria, a photo-induced effect was observed. These five eudistomins (**45**–**47**, **50**, and **51**) were active or more significantly active under ultraviolet A (UVA) exposure against murine cytomegalovirus, *Sindbis virus*, *S. cerevisiae, S. albus, B. subtilis, E. coli,* and mammalian 3T3 cells [96]. The antibacterial, antifungal, and antiviral effects were indeed not correlated, suggesting different mechanisms of action against different microorganism, depending upon the presence or absence of UVA [96].

Eudistomins W and X (**52** and **53**, Figure 8) were obtained from a Micronesian *Eudistoma* sp. [97]. Compound **53** showed antibacterial activity against *B. subtilis* (17 and 18 mm of inhibition zones), *S. aureus* (11 and 12 mm), and *E. coli* (15 and 20 mm) at loading doses of 5 and 10 μg per disk, respectively, and fungicidal against *C. albicans*, with inhibition zones of 17 and 18 mm at similar loading doses, too. On the contrary, eudistomin W (**52**) was selectively active toward *C. albicans*, giving a zone of inhibition of 13 mm at 10 μg.

Further, eudistomins (eudistomin Y1–Y7, **54**–**60,** Figure 8) have been isolated from a South Sea collection of *Eudistoma sp.* [91]. In this study, eudistomin Y6 (**59**) was shown to be moderately active against *S. epidermis* and *B. subtilis*. Eudistomins Y2–Y7 (**55**–**60**) were re-isolated from the Korean ascidian *Synoicum* sp., together with other derivatives, eudistomins Y8–Y13 (**61**–**66**, Figure 8) [93]. Eudistomin Y10 (**63**) showed a potent inhibition against various bacterial strains such as *B. subtilis* (12.5 µg/mL) and *Proteus vulgaris* (12.5 µg/mL). Moreover, eudistomin Y7 (**60**) showed anti-fungal activity on *Aspergillus fumigatus* (50 µg/mL) and *Trichophyton rubrum* (50 µg/mL).

7-Bromo-*N*-hydroxyhomotrypargine (**67**, Figure 8) was isolated from the New Zealand ascidian *P. opacum* [57]. It exhibited significant activity toward *S. aureus* in microbroth-dilution assays with MIC and MBC values of 32 μg/mL (53 μM), but was less active against *E. coli* (MIC 64 μg/mL, MBC > 64 μg/mL [106 μM]) and inactive toward *M. tuberculosis* BSG001 (MIC and MBC > 64 μg/mL [106 μM]). In addition, compound **67** exhibited a moderate antimalarial activity; in particular, it was tested against a chloroquine-resistant strain (FcB1-Colombia) of *Plasmodium falciparum* and found to exhibit an IC_50_ value of 3.8 μM. Thus, the *β*-carboline system is an additional example of a new marine derived chemical scaffold useful for development of antimalarial agents [40,41,98,99,100,101].

Didemnolines A–D **68**–**71** (Figure 8) were isolated from the ascidian *Didemnum* sp., collected in Northern Mariana Islands (USA) [92]. When tested against several bacterial strains, only didemnoline C (**70**) showed 7 mm and 9 mm growth inhibition zones against *E. coli* and *S. aureus*, respectively [92].

**Figure 9:**
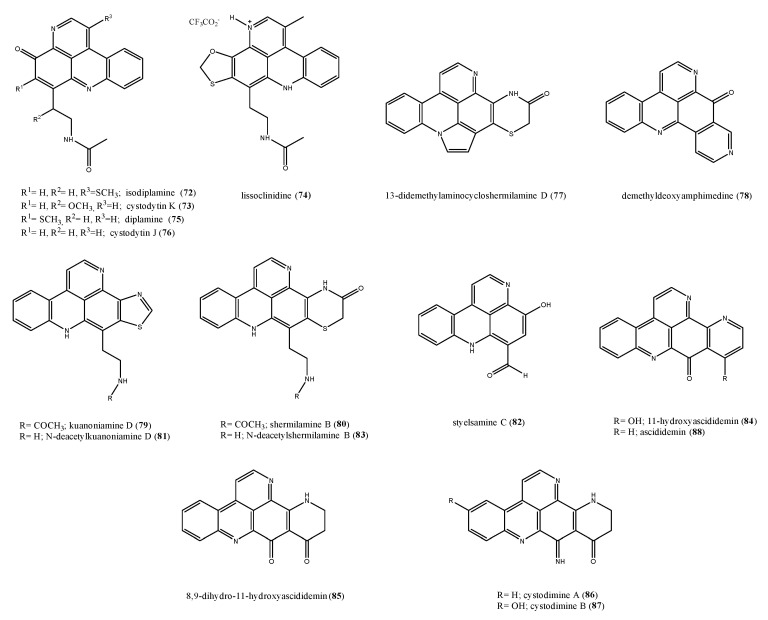
Structures of the pyridoacridine alkaloids **72**–**88**.

Pyridoacridines isolated from ascidians are typically tetra- or penta-cyclic aromatic alkaloids based on the pyrido[k,l]acridine skeleton with a broad range of bioactivities including cytotoxic, antibacterial, antifungal, antiviral, insecticidal, anti-HIV, and anti-parasitic [88,102,103]. Examples of antimicrobial piridoacridines are isodiplamine (**72**), cystodytin K (**73**), lissoclinidine (**74**), diplamine (**75**), and cystodytin J (**76**) (Figure 9) from *Lissoclinum notti* [104], active towards a variety of microorganisms, including the bacteria *B. subtilis* and *E. coli* and the fungi *C. albicans* and *Trichophyton mentagrophytes*. Several antimicrobial pyridoacridine alkaloids have been reported from *Cystodytes dellechiajei*, a colonial soft-bodied ascidian widely distributed in tropical and temperate waters, which occurs in a range of colour morphs [105,106,107,108,109,110,111]. Interestingly, chemical investigation of ascidian different color morphs yielded different piridoacridine structures. Bry et al. reported the isolation from a purple chromotype from Mediterranean sea of 13-didemethylaminocycloshermilamine D (**77**) and demethyldeoxyamphimedine (**78**) (Figure 9) [112]. Both pyridoacridines (**77** and **78**) were tested against the marine bacterial strain *L. anguillarum* (7.0 ˂ MIC value range ˂ 9.0 μM) and the terrestrial bacterial strain *M. luteus* (6.5 ˂ MIC value range ˂ 7.0 μM), showing an activity against the two strains in the order of micromolar with less activity on the marine strain. A purple colour morph collected in Catalonia afforded kuanoniamine D (**79**), shermilamine B (**80**), *N*-deacetylkuanoniamine D (**81**), styelsamine C (**82**), and *N*-deacetylshermilamine B (**83**) [113,114,115,116]. Some green colour morphs yielded 11-hydroxyascididemin (**84**) [108,109], 8,9-dihydro-11-hydroxyascididemin (**85**) [117], cystodimine A (**86**), and cystodimine B (**87**) [107], while, from the extract of a blue morph, only ascididemin (**88**) was obtained (Figure 9) [106,107]. Ascididemin was found to be active against *E. coli*, *Cladosporium resinae*, and *B. subtilis*, but inactive toward *P. aeruginosa* and *T. mentagrophytes* [118]. Each compound was tested against two bacterial strains *E. coli* and *M. luteus* and a dose-dependent inhibition was observed for all tested pyridoacridines against the two strains. The MIC values ranged from 0.2 to 2.6 µM toward *E. coli* and from 0.3 to 17.4 µM toward *M. luteus*.

**Figure 10:**
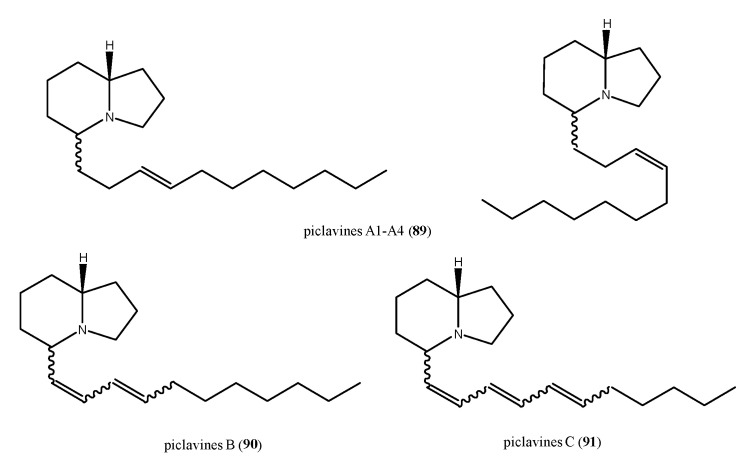
Structures of the indolizine alkaloids piclavines (**89**–**91**).

Piclavines A–C (**89**–**91**, Figure 10) are isomeric indolizidine alkaloids isolated from the ascidian *Clavelina picta* [119]. Piclavines differ in the number of double bonds on the side chain and each group is composed of an inseparable mixture of stereoisomers, which differ for both the configuration at C2 and geometry at the double bond. The piclavines A–C are the first indolizidines isolated from a marine source; they exhibited a pronounced antifungal and antimicrobial activity against Gram-positive bacteria (*C. albicans*, *Geotrichium candidum*, *A. terreus*, *S. aureus*, *B. cereus*, *Corynebacterium michiganensis*), while none of the Gram-negative bacteria tested (*E. coli*, *P. aeruginosa*, and *X. campestrum*) showed any response toward these indolizidines.

**Figure 11:**
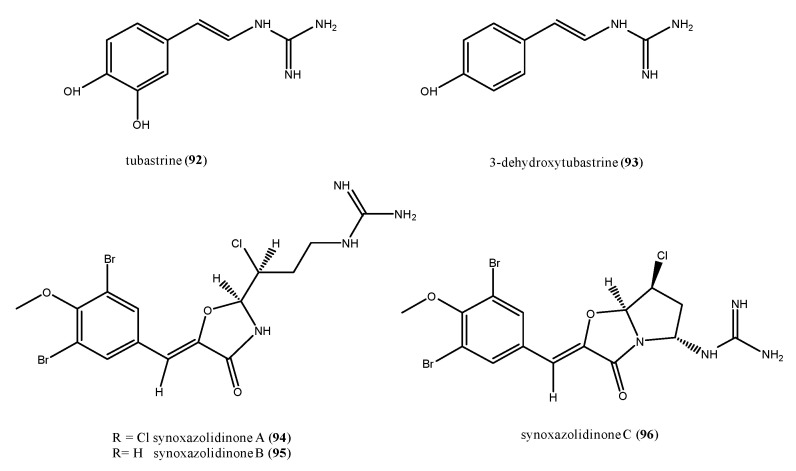
Structures of the guanidine alkaloids tubastrine (**92**), 3-dehydroxy tubastrine (**93**), and synoxazolidinones A–C (**94**–**96**).

Marine natural products incorporating a guanidine functionality are not uncommon, and often exhibit interesting biological activities [120]. An example is tubastrine (**92**), firstly isolated from a coral [121], but successively found in the ascidians *Dendrodoa grossularia*, *Ascidiella scabra*, and *Aplidium orthium* (Figure 11) [122,123]. Tubastrine (**92**) showed antiviral activity against *Herpes simplex* virus type I and vesicular stomatitis virus [121], besides anticancer and anti-inflammatory activity [123]. 3-Dehydroxytubastrine (**93**) is a closely turbastrine-related compound isolated from the sub-Arctic ascidian *Dendrodoa aggregate* [124]. It showed antimicrobial activity against several bacteria such as *Corynebacterium glutamicum*, *S. aureus*, and methicillin-resistant *S. aureus* (MRSA), at a concentration of 100 µg/mL [124], as well as against *Serratia* (500 µg/disk) and *E. coli* (100 µg/disk) [125].

Synoxazolidinones A–C (94–96, Figure 11) form a family of dibrominated guanidines containing a unique 4-oxazolidinone core structure, which were isolated from *S. pulmonaria* collected off the Norwegian coast [126,127]. Synoxazolidinone A (94) displayed activity against the Gram-positive bacteria *S. aureus*, MRSA, with MIC values of 10 μg/mL each, and against *C. glutamicum* with an MIC value of 6.25 μg/mL. It was also effective against *S. cerevisiae* (MIC = 12.5 μg/mL). Synoxazolidinone B (95) was less potent than synoxazolidinone A (94) against MRSA with an MIC of 30 μg/mL; this suggested that the chlorine atom is important for the biological activity [126]. Synoxazolidinone C (96) exhibited the same antibacterial potency of synoxazolidinone A (94) on *S. aureus* and MRSA (MIC =10 μg/mL each) and was active also against *E. coli* and *E. faecalis* at concentrations of 30 and 20 µg/mL, respectively [127].

### 2.7. Furanones and Other Brominated Aromatic Derivatives

Rubrolides and cadiolides are two families of structurally related halogenated aromatic compounds endowed with a central furanone ring isolated from different marine ascidians such as *Ritterella*, *Pseudodistoma*, *Botryllus*, and *Synoicum* [128,129,130,131,132,133]. Two rubrolides have also been found into a culture of the fungus *Aspergillus terreus* [134]. These compounds possess a number of bioactivities, including anti-inflammatory, cytotoxic, as well as inhibitory activity against protein phosphatases. Moreover, both these groups of furanone derivatives may be considered as two interesting classes of natural antibiotics.

**Figure 12:**
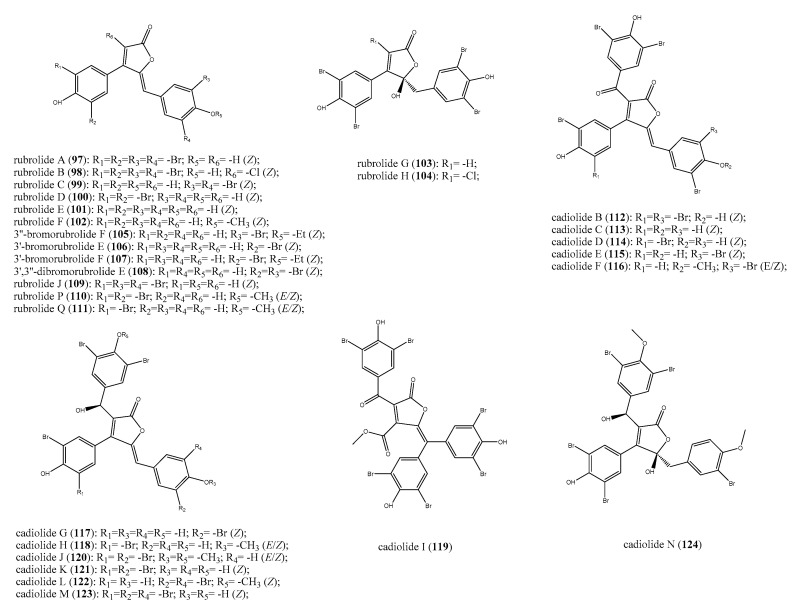
Structures of the furanone derivatives rubrolides A–H, J, P, and Q (**97**–**111**) and cadiolides B–N (**112**–**124**).

The first report of antibacterial effects exhibited by rubrolides dates from the biological evaluation of organic extracts from *Ritterella rubra* specimens [128]. The bioassay-guided fractionation of these extracts led to the identification of rubrolides A–H (**97**–**104**, Figure 12) as responsible for the antibacterial effects against *S. aureus* and *B. subtilis*. The most active compounds in the series were rubrolides A–C (**97**–**99**), which exhibited MIC values of 9, 2, and 11 μg/disk against the two strains, respectively. Rubrolides E and F (**101** and **102**) were re-isolated from samples of *Synoicum sp.* collected in South Africa area along with the strictly related compounds 3″-bromorubrolide F (**105**), 3′-bromorubrolide E (**106**), 3′-bromorubrolide F (**107**), and 3′,3″-dibromorubrolide E (**108**) (Figure 12) [132]. The possibility for **101**–**102** and **105**–**108** to act against a panel of dangerous bacteria such as MRSA and *S. epidermidis* as well as gentamycin- and vancomycin-resistant *E. faecalis* and *E. coli* strains was investigated. Rubrolide F (**102**) and its brominated derivatives **105** and **107** were rather ineffective against all the tested bacteria, whereas rubrolide E (**101**) and its analogues **106** and **108** showed different effects. Despite being weakly active against MRSA (IC_50_ = 105 μM), rubrolide E was the most active compound against *S. epidermidis* with an IC_50_ was of 21 μM, followed by 3′,3″-dibromorubrolide E (**108**, IC_50_ = 28 μM) and 3′-bromorubrolide E (**106**, IC_50_ = 38 μM). Additionally, **101** exhibited the higher percentage (89%) of *E. faecalis* growth inhibition, whereas it was less potent against *E. coli* (16% of inhibition growth). Its dibrominated derivative (**108**), instead, was the most potent in the series against *E. coli* with 26% of growth inhibition with respect to all other compounds, mainly the 3’-bromorubrolide E (**106**), which was totally inactive against this strain [132].

Rubrolides J (**109**), P (**110**), and Q (**111**) (Figure 12) were obtained from the ascidian *P. antinboja* [130]. They possessed a weak or moderate antibacterial activity against several Gram-positive bacteria, but they were inactive against Gram-negative strains. Rubrolides P and Q (**110** and **111**) were inactive against the drug-resistant bacteria strains, and indeed, their IC_50_ values (μg/mL) were always greater than the cut-off. Nevertheless, they showed some toxic effects only against the Gram-positive actinobacteria *Kocuria rhizophila* against, which exhibited IC_50_ of 6.3 and 3.1 μg/mL, respectively. More interesting was rubrolide J (**109**), which had IC_50_ of 0.8 and 1.3 μg/mL against *S. epidermidis* and *B. subtilis*, respectively, and was not toxic against mammalian cells. Rubrolides A and J drew attention as their antibacterial efficacy was comparable to or even better than linezolid and platensimycin when evaluated against several methicillin-sensitive and -resistant *S. aureus* strains [130].

Cadiolides are a family of ascidians secondary metabolites structurally related to rubrolides; they share the same 3-aryl-4-arylmethylenefuranone unit and cadiolides bring up an additional 2-ketoaryl substituent and a different carbon backbone. The family of cadiolides is represented by compounds named from A to N; apart from cadiolide A, the first discovered compound of this class from ascidians of the genus *Botryllus* for which any bioactivity has not been reported up to now [129], all the remaining cadiolides B–N (**112**–**124**, Figure 12) showed antibacterial properties. They have been isolated from specimens of *Botryllus* sp.*, P. antinboja*, and *Synoicum* sp. and their biological effects have been investigated [129,130,131,133,135]. Cadiolides B–N (**112**–**124**) were tested against several strains of both Gram-positive and negative bacteria as well as drug-resistant strains; the obtained results evidenced some structural requirements needed for the bioactivity, as described below. Compounds **112**–**124** did not show any effect on the growth of Gram-negative bacteria such as *E. coli*, S*. typhimurium*, and K*. pneumoniae*, as well as against the Gram-positive *E. faecalis* and *E. faecium*. Only cadiolide L (**122**) inhibited the growth of *E. faecalis* in vitro, showing an MIC of 8 μg/mL [135].

The antibacterial properties of cadiolides B–N against an array of bacteria strains with related MIC values are reported in Table 2; these data underline their promising potential to be a new class of antibiotics worthy of further investigation. It has been suggested that the hydroxyl group located at C-6 might be more necessary for the antibacterial activity than the ketone group, but not enough experimental evidence has been obtained to support this hypothesis. Surely, the methylation of the phenolic moiety caused an overall reduction of activity against all the tested strains, as demonstrated, for example, for cadiolides F, J, and N when compared with cadiolides C, E, or I. Moreover, the number and the position of bromine atoms is important in term of bioactivity. Generally, cadiolides were antibacterial agents with IC_50_ values better than or comparable to those of rubrolides. Thus, an additional polysubstituted aromatic ring linked at C-2 may be beneficial to assess the antibacterial potential [130,131,133]. Therefore, the furanone moiety described for these classes of marine natural antibiotics has been recognised as a new active chemotype for the development of more selective and potent antibacterial derivatives considering the novelty of this scaffold with respect to the benchmark antibiotics [130].

**Figure 13:**
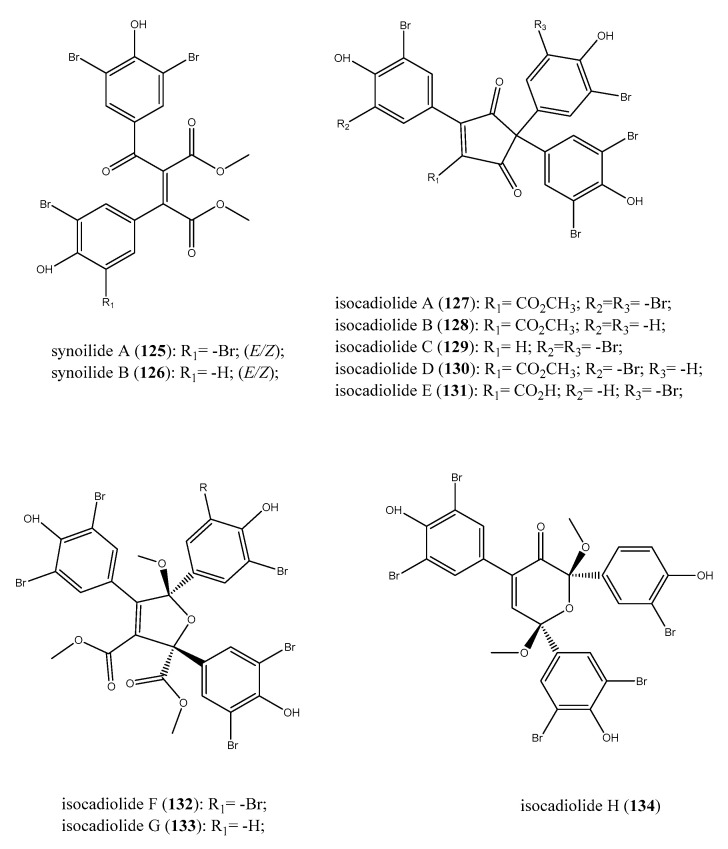
Structures of synoilides A (**125**) and B (**126**) and isocadiolides A–H (**127**–**134**).

Several metabolites originating from rearrangements and oxidization of the furanone core of rubrolides/cadiolides in different extents have been discovered. Synoilides A (**125**) and B (**126**) are two bis-aromatic esters related to rubrolides (Figure 13). These halogenated compounds are characterized by an unreported carbon skeleton that offered clear information of the structure–activity relationship for these metabolites endowed with a furanone and/or furanone-like portion [131]. Indeed, synoilides A and B exhibited only a weak antibacterial effect against *B. subtilis* and *S. enterica* (MICs of 50 μg/mL) and, for this reason, the furanone ring appeared to be crucial for the bioactivity.

Further studies of *Synoicum* sp. ascidian offshore of Keomun-do Island in Korea afforded the isolation of isocadiolides A–H (**127**–**134**), likely produced by rearrangements followed by oxidization of the furanone ring. These compounds are polybrominated aromatics that possess a tris-bromohydroxyphenil moiety linked to varied cores such as a cyclopentenedione, a dihydrofuran, or a pyranone ring (Figure 13) [135]. Chemical modification of the structural core determined a significant decrease of bioactivity; all isocadiolides exhibited a weaker activity than the relevant cadiolides. Among compounds **127**–**134**, isocadiolide C (**129**) was the most potent against *S. aureus*, with an MIC of 4 μg/mL, together with isocadiolides A, B, and D (**127**, **128**, **130**; 16 < MIC (μg/mL) < 32); the Gram-positive *E. faecalis* was significantly compromised by isocadiolides G (**133**), which had MIC = 8 μg/mL, whereas isocadiolide B (**128**) possessed the same MIC against *P. hauseri*.

Although less potent than cadiolides, isocadiolides A–D (**127**–**130**) were effective in the inhibition of the transpeptidase sortase A of *S. aureus,* the enzyme involved in the virulence of Gram-positive bacteria (IC_50_ in the range 67–102 μM) [136]. These results were particularly encouraging as the positive control used, berberine chloride, had an IC_50_ of 102 μM [135]. Analogously, compounds **127**–**130** also showed similar values against the key enzyme isocitrate lyase of *C. albicans,* with IC_50_ of isocadiolide A (22 μM), comparable to that of positive control 3-nitropropionate (17 μM) [137].

### 2.8. Exploring the Antimicrobial Properties of Metabolites from Ascidian-Associated Microorganisms

A rich and filled community of microbes has been discovered to be associated with ascidians taking an active part in vital and metabolic functions of the marine invertebrates [23]. The impact of the natural products on the global health for the treatment of an array of diseases led to an extensive analysis of the biogenetic pathways, shedding light on their real producers. Hence, potent bioactive secondary metabolites, first considered to be synthesised by the ascidians, have later been further studied to assess their microbial origins, and thus the rich ascidian microbial diversity [138,139,140]. The well-known antitumor compound ecteinascidin 743 or the antineoplastic depsipeptide didemnin B, for example, are actually biosynthesised by ascidian-associated microorganisms, which have been defined as *Candidatus* Endoecteinascidia frumentensis, and *Tistrella mobilis* and *T. bauzanensis*, respectively [141,142]. Moreover, several studies have also demonstrated that the pairing ascidian-microorganism is species-selective [24,138,143]. Thus, in this last section of the present review, we describe several molecules isolated by ascidian-associated microorganisms and/or metabolites for which microorganisms have been established as their real producers.

#### 2.8.1. Ascidians-Associated Actinobacteria as Producers of Antimicrobial Compounds

**Figure 14:**
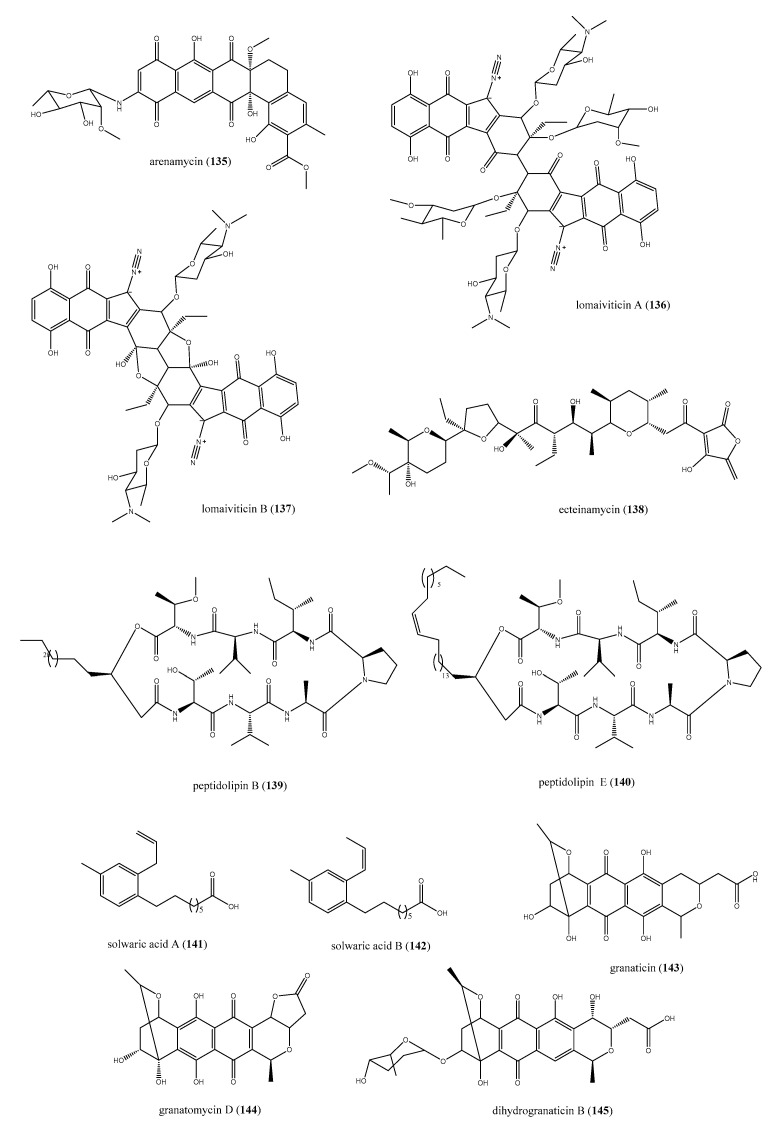
Structures of compounds **135**–**145** derived from ascidian-associated actinobacteria.

The actinobacteria *Salinospora arenicola* (CNR-647), associated with the ascidian *Ecteinascidia turbinata*, afforded arenimycin (**135**, Figure 14), a benzo[α] naphtacene quinone compound with significant antibiotic properties [144]. Arenimycin (**135**) exhibited a strong inhibition of bacterial growth with a MIC < 1 μg/mL against either multi-drug resistant *Staphylococcus* strains or *E. faecalis* and *faecium*. Moreover, arenimycin showed MIC = 1 μg/mL against the *M. bacille*, too. However, it was also active against human adenocarcinoma cell line (HCT-116) with IC_50_ = 1.16 μg/mL, thus these cytotoxic effects indicate a non-selective mechanism of action prevented further exploitation on this class of antibiotic compounds [144].

Another strain of actinobacteria of genus *Salinospora*, *S. pacifica*, was identified as the real producer of two diazotetrahydrobenzo[b]fluorene glycosides, lomaiviticins A and B (**136** and **137**, Figure 14) [145,146], isolated the ascidian *P. lithostrotum*. Initially, the two compounds were supposed to be produced by fermentation of a halophilic strain of the actinomycete *Micronospora lomaivitiensis* [145]. Subsequently, Janso and co-workers were able to identify the gene cluster of biosynthesis of compounds **136** and **137** by genome sequencing. This gene was related to *S. pacifica* strains DPJ-0016 and DPJ-0019, an actinobacteria with symbiotic association with the above-mentioned tunicate, too. Both compounds caused a potent DNA damage with a cleavage of the double stranded DNA, whereas lomaiviticin A (**136**) had a cytotoxicity profile comparable to DNA-damaging anticancer drugs, like adriamycin and mitomicin C. Alongside these effects, for two glycosides, the antibacterial activities were assessed. In particular, they were effective against *S. aureus* and *E. faecium,* with MICs ranging from 6 to 24 ng per spot [145].

The marine *Actinomadura sp.* strains, associated with *E. turbinata*, were recognized as the producers of ecteinamycin (**138**, Figure 14) [147]. Ecteinamycin (**138**) showed an excellent selectivity for *Clostridium difficile* NAP1/B1/027 against that possessed an MIC of 59 ng/μL. Further studies for its mechanism of action, based on chemical genomics, highlighted, for **138**, a high selectivity for H^+^/K^+^ ATPase disrupting vesicular trafficking, thus compromising the effects of *Clostridium* toxins [147].

The marine-derived *Nocardia sp.* has been demonstrated as a prolific source of secondary metabolites with various therapeutic applications including antibiotics [148]. The cultivation of *Nocardia sp.* WMMB215 strain, isolated from the ascidian *Trididemnum orbiculatum*, was found to produce several lipopeptides strictly correlated to the *L*-Val analogue of peptidolipin NA [149]. These compounds were correlated owing to a peptide cyclized via an ester to a lipophilic tail; some of these are discussed here seeing their antibacterial properties. The 80% mortality at 30 days of infection is caused by MRSA [150,151]. Hence, it is clearly necessary to discover new agents acting in this way. Focusing on this bioactivity, peptidolipins B (**139**) and E (**140**) from *Nocardia sp.* reported in Figure 14 are examples of compounds that inhibit methicillin-resistant *S. aureus* (MRSA) and methicillin-sensitive *S. aureus* (MSSA) strains. Interestingly, they showed a moderate antibacterial effect, considering an MIC of 64 μg/mL for both. Peptidolipins B and E were also investigated for the putative bacteriostatic, rather than bactericidal, mechanism of action. The wells, in which the bacterial growth was inhibited, were treated with a sterile swab and, as bacteria resumed to grow, **139** and **140** were deemed as bacteriostatic agents [149].

Two trialkyl-substituted aromatic acids, solwaric acids A and B (**141** and **142** reported in Figure 1), are further compounds isolated from an ascidian-associated microorganism, the actinobacteria *Solwaraspora sp.* [152]. This microbe such as *Nocardia sp.* was identified from *T. orbiculatum* and was the producer of metabolites such as **141** and **142**. Both resulted in the inhibition of bacterial growth in vitro and, interestingly, were more potent against MRSA than MSSA strains, showing MIC = 32 μg/mL against the methicillin resistant bacteria versus MIC = 64 µg/mL against MSSA [152].

*Streptomyces* spp. are a renowned reservoir of chemically diverse and biologically active metabolites, and have well adapted to living associated to marine ascidians [148,153]. The search of antimicrobial compounds from the solitary brown ascidian *Styela canopus* allowed the isolation of granaticin (**143**), granatomycin D (**144**), and dihydrogranaticin B (**145**), whose production was related to a strain of *Streptomyces* (PTY087I2) associated with the ascidian (Figure 14) [154]. Interestingly, the presence of compounds **143**–**145** in the co-cultures of PTY087I2 with MRSA strains enhanced the antibacterial efficacy; indeed, this co-culture was 16-, 4-, and 8-fold more active against *B. subtilis*, MSSA, and MRSA, respectively [154].

#### 2.8.2. The Role of Fungi in the Biosynthesis of Antimicrobial Compounds

**Figure 15:**
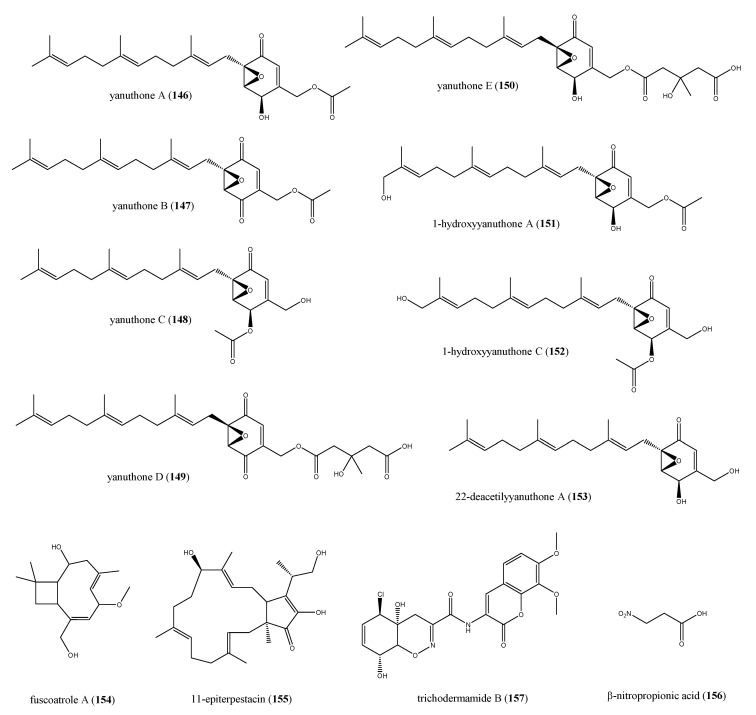
Structures of compounds **146**–**157** derived from ascidian-associated fungi.

Fungi from marine environment are the second major class of ascidian-associated microorganisms, producers of secondary metabolites with an increased antimicrobial potential with respect to those derived from terrestrial fungi [155]. The production of several ascidian-derived bioactive compounds has been ascribed to *Aspergillus sp*., as demonstrated by the chemical analysis of marine fungal isolates from ascidians [156,157]. The symbiotic association between *A. niger* and the orange tunicate *Aplidium* sp. yielded a series of farnesylated epoxy cyclohexenones active against varied microorganism strains. The presence of quinone and quinone-derived moieties in marine metabolites is owing to the shikimic acid pathway supposed to be present in microbes and lacking in animals [158]. The *Aplidium*-associated *Aspergillus* was involved in the biosynthesis of yanuthones A-E (**146**–**150**), 1-hydroxyyanuthone A (**151**), 1-hydroxyyanuthone C (**152**), and 22-deacetylyanuthone A (**153**) (Figure 15). All compounds **146**–**153**, except for yanuthones D and E (**149** and **150**), displayed only a slight inhibition of bacterial growth, with inhibition zones ranging from 8 to 10 mm against MSSA, MRSA, and vancomycin-resistant *Enterococcus sp.* when tested with the agar diffusion assay. Yanuthones D and E (**149** and **150**) were the most active compounds in the series, probably owing to the presence of a hydroxymethylglutaril (HMG) group at position 22, which increase the polarity of molecules, allowing a better agar diffusion and, definitely, a higher antibacterial effect. Yanuthone D showed zones of inhibition of 15 mm against either MSSA and vancomycin-resistant *Enterococcus sp.*, while these zones for yanuthone E were of 13 and 12 mm, respectively. Interestingly, both compounds were more potent against MRSA, exhibiting an inhibition zone of 17 mm at 62 μg for **149** and 100 μg for **150** [158].

Fuscoatrole A (**154**), the bicyclic sesterpene 11-epiterpestacin (**155**), and β-nitropropionic acid (**156**, Figure 15) were extracted from *Humicola fuscoatra* (KMM4629 strain), symbiont of undefined Kurial colonial ascidian [159]. Fuscoatrole A (**154**) is the first example of caryophyllenic sesquiterpene isolated from marine fungi, because caryophyllene type compounds are peculiar of terrestrial plants and fungi [160,161,162]. All three compounds **154**–**156** were effective against *S. aureus* and *B. subtilis*, with an MIC = 12.5 μg/mL for fuscoatrole A, whereas **145** and **146** showed only a weak effect (MIC = 100 μg/mL). Therefore, β-nitropropionic acid (**156**) was also able to prevent both *C. albicans* and *E. coli* growth in vitro and its MIC value was calculated to be equal to 100 μg/mL [159].

*Trichoderma virens* is a species of ascomycete, and its association with many ascidians of genus *Didemnum* has been discovered. This fungi strain is noted for the production of the potent antibiotic gliotoxin, although it has been dismissed owing to its severe toxic effects in vivo [163]. The antibiotic trichodermamide B (**157**, Figure 15) was obtained from a culture of *T. virens* isolated from the ascidian *D. molle* collected along the cost of Papua New Guinea [164]. The X-ray diffraction permitted to define its structure that was characterized by the presence of a rare cyclic *O*-alkyl-oxime group into a six-membered ring. Compound **157** exhibited an MIC of 15 μg/mL against amphotericin-resistant strains of *C. albicans*, MRSA, and vancomycin-resistant strains of *E. faecium*, beyond a significant cytotoxic effect against HCT-116 cell line (IC_50_ = 0.32 μg/mL). The bioactivity of trichodermamide B (**157**) might be related to the chlorohydrin moiety, which could lead to an epoxide ring, which could represent its biologically active form [164].

#### 2.8.3. Bisanthraquinones from Cyanobacteria

**Figure 16:**
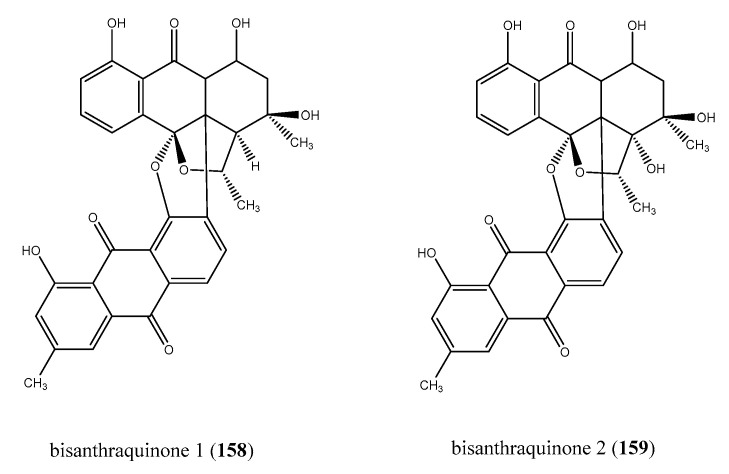
Structures of the cyanobacteria-derived bisanthraquinones 1 and 2 (**158** and **159**).

Nowadays, the role of cyanobacteria as producers of a wealth number of natural products is ever more emerging, helping to account for the symbiosis between microorganisms and marine invertebrates (Figure 1A) [137]. Cyanobacteria represent a class of photosynthetic Gram-negative bacteria also called blue-green algae, one of the oldest forms of life on Earth [165]. Harmful blooms of marine cyanobacteria are increasing as a consequence of human population growth as well as agricultural, urban and industrial activities. [166,167]. Nevertheless, cyanobacteria are producers of structurally diverse metabolites endowed with a broad spectrum of biological properties [168,169]. Bisanthrantaquinones 1 (**158**) and 2 (**159**) have been isolated from the blue-green algae strain (URI strain # N36-11-10) identified from the colonial ascidian *E. turbinate* and are the known antibacterial metabolites from ascidian-associated cyanobacteria, up to now (Figure 16) [170]. Bisanthrantaquinone 1 (**158**) showed a greater antibacterial effectiveness than 2 (**159**); indeed, **158** exhibited MICs of 0.15 and 2.0 μM against MRSA and vancomycin-resistant *E. faecalis* with respect to MICs of 0.36 and 12 μM shown by **159**, respectively. Bisanthraquinone 1 (**158**) was altogether 10-fold more active against MRSA than vancomycin-resistant strain of *E. faecalis* [170].

## 3. Conclusions

The present review describes about 160 molecules endowed with antimicrobial activity produced by ascidians and/or by their associated microorganisms. The high and intrinsic chemiodiversity of ascidian substances is remarkable and strongly affords a chemical reservoir of peculiar scaffolds. This chemical and biological diversity represents a great opportunity to meet the urgent and increasing need of new antibiotic agents. The unique chemical class of ascidians antimicrobial metabolites, including sulfur-containing compounds, meroterpenes, alkaloids, peptides, furanones, and other aromatic derivatives, as well as their antimicrobial effects, undoubtedly could provide lead candidates for drug discovery programs in the anti-infective area of interest. The bioactivity of several metabolites is highly meaningful if compared with currently marketed drugs. Among these, lissoclinotoxin A (**1**) exhibits a very low MIC value (0.08–0.15 μg/mL) in the same range of the commercial cefatoxim against *S. aureus*. Analogously, namenamicin (**7**), the only enediyne natural product of marine origin, was a potent antibiotic, even more active than penicillin G against a wide panel of bacteria and fungi (MICs ranging 0.004–0.25 μg/mL). Furthermore, the strong antifungal potency against *C. albicans* (MIC = 0.7 ± 0.05 μg/mL) and the moderate effect against *C. glabrata* (MIC = 30.0 μg/mL) allow to consider the (2*S*, 3*R*)-2-aminododecan-3-ol (**34**) to be comparable to clinically used antifungal agents, such as nystatin and ketoconazole. Interestingly, the antibacterial efficacy of rubrolide A (**97**) and J (**109**) was comparable or even better than two antibiotic marketed-drugs, linezolid and platensimycin, against MSSA and MRSA strains. Therefore, although the ascidian′s secondary metabolites have been highlighted as an extraordinary arsenal of novel drug lead structures, the studies have often been limited to the knowledge of the biological effects in vitro. Very few attempts, not to say none, have been made to deepen the pharmacological characterization of these molecules, for example, by investigating the mode of action of the active metabolites; in some cases, simple structure activity relationships (SARs) have been tentatively proposed. This fact, if on the one hand can be considered a disadvantage, on the other shows us, in perspective, a still virgin and practically unexplored field of exploration of new structures and new mechanisms, which could play an important role in the future in the fight against the global infectious-disease burden and the resistance issue.

## Figures and Tables

**Table 1:** Minimum inhibitory concentration (MIC) values expressed in μg/mL of pseudoaminols A (**27**) and B (**28**) against bacteria and human cancer cell lines.

Bacterial Strains	MIC (μg/mL) ^a^		Mammalian Cell Line	LC_50_ (μM) ^b^	
	27	28		27	28
*S. aureus*	12.5	12.5	A549	13.8	13.1
*B. subtilis*	25	25	K562	13.6	12.6
*M. luteus*	12.5	12.5			
*S. typhimurium*	6.25	12.5			
*P. vulgaris*	25	25			
*E. coli*	>100	>100			

^a^ Ampicillin has been used as positive control with MIC of 0.4 μg/mL against *S. aureus*, *B. subtilis*, *M. luteus*, and *S. typhimurium*; 1.6 μg/mL against *P. vulgaris*; 6.3 μg/mL against *E. coli*. ^b^ Doxorubicin as positive control with LC_50_ (μM) of 0.9 (A549) and 1.1 (K562).

**Table 2:** MIC values (μg/mL) of cadiolides B–N (**112**–**124**).

Compounds	MIC (μg/mL)				
	A ^a^	B ^a^	C ^a^	D ^a^	E ^a^
cadiolide B (**112**)	3.1	3.1	3.1	>128	>128
cadiolide C (**113**)	0.4	0.2	3.1	64	>128
cadiolide D (**114**)	6.3	1.6	6.3	>128	>128
cadiolide E (**115**)	3.1	0.2	1.6	1.6	3.1
cadiolide F (**116**)	12.5	3.1	12.5	- ^b^	- ^b^
cadiolide G (**117**)	3.1	3.1	12.5	0.8	3.1
cadiolide H (**118**)	6.3	3.1	1.6	3.1	3.1
cadiolide I (**119**)	0.8	0.8	0.8	1.6	6.3
cadiolide J (**120**)	32	- ^b^	- ^b^	64	8
cadiolide K (**121**)	8	- ^b^	- ^b^	8	4
cadiolide L (**122**)	2	- ^b^	- ^b^	4	2
cadiolide M (**123**)	1	- ^b^	- ^b^	- ^b^	- ^b^
cadiolide N (**124**)	16	- ^b^	- ^b^	16	16

^a^ A: *S. aureus*; B: *K. rhizophila*; C: *B. subtilis*; D: *S. enterica*; E: *Proteus hauseri*; ^b^ the symbol “-” means that the compound has not been tested yet.
